# Eosinophilic granulomatosis with polyangiitis developed during treatment with benralizmab for severe asthma: A case report and literature review

**DOI:** 10.1002/rcr2.1431

**Published:** 2024-07-09

**Authors:** Mitsukuni Sakabe, Kazunori Tobino, Yumi Obata, Shota Sogabe, Kazuki Uchida, Yosuke Murakami

**Affiliations:** ^1^ Department of Respiratory Medicine Iizuka Hospital Iizuka Japan; ^2^ Department of Respiratory Medicine Juntendo University, School of Medicine Bunkyo‐Ku Japan

**Keywords:** asthma, benralizumab, EGPA, mepolizumab, purpura

## Abstract

Eosinophilic granulomatosis with polyangiitis (EGPA) is a rare autoimmune disorder characterized by necrotizing vasculitis, asthma, and eosinophilia. We report a case of EGPA that developed during benralizumab treatment for severe asthma and provide a literature review. A 79‐year‐old Japanese male with severe asthma presented with generalized purpura 4 months after initiating benralizumab treatment. He had reduced his oral prednisolone dose from 7.5 to 2 mg/day. Laboratory tests revealed eosinophilia, and skin biopsy showed vasculitis with eosinophilic infiltration. He was diagnosed with EGPA and treated with corticosteroids, azathioprine, and mepolizumab, which led to rapid improvement and sustained remission. Five cases of EGPA developing during benralizumab treatment have been reported, with onset ranging from 14 to 36 weeks after initiation. Clinicians should monitor for EGPA development in patients receiving benralizumab, particularly during oral corticosteroid reduction.

## Graphical abstract

We present a case of eosinophilic granulomatosis with polyangiitis (EGPA) that developed during treatment with benralizumab for severe asthma and provide a comprehensive literature review to further our understanding of this rare and challenging condition.

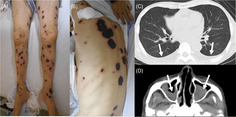

## INTRODUCTION

Eosinophilic Granulomatosis with Polyangiitis (EGPA), formerly known as Churg‐Strauss syndrome, is a rare autoimmune disorder characterized by necrotizing vasculitis affecting small to medium‐sized blood vessels. EGPA typically manifests in three phases: adult‐onset asthma with sinusitis and nasal polyposis; peripheral blood eosinophilia leading to systemic manifestations; and necrotizing vasculitis affecting multiple organ systems.^1^, ^2^ Until recently, treatment consisted of corticosteroids and immunosuppressive agents. However, targeted biologics such as mepolizumab have revolutionized EGPA management by targeting interleukin‐5 (IL‐5), effectively reducing eosinophil levels and inflammatory processes.^3^, ^4^ Similarly, benralizumab, a monoclonal antibody that targets the IL‐5α receptor, has also been reported to be effective in the management of EGPA.^5^ Although promising, there have been reports of EGPA developing during benralizumab treatment for severe asthma, highlighting the need for continued vigilance and surveillance. In this report, we present a case of EGPA that developed during treatment with benralizumab for severe asthma and provide a comprehensive literature review to further our understanding of this rare and challenging condition.

## CASE REPORT

**FIGURE 1:**
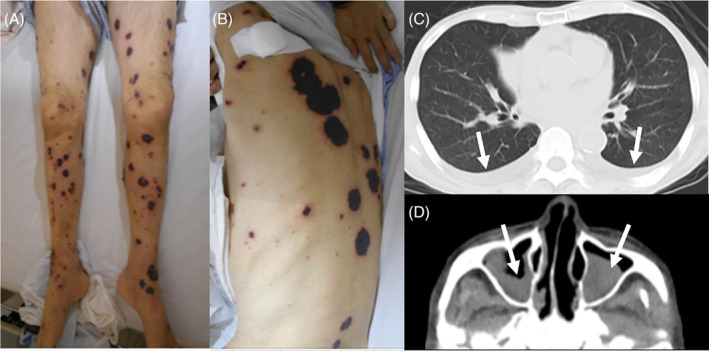
Findings of our case on admission. (A,B) The patient presented with numerous purpuric lesions accompanied by erythema and hematomas distributed on the extremities and trunk. (C) Bilateral pleural effusion was observed on chest CT. No other significant findings were noted (white arrows). (D) Head CT revealed mucosal thickening in the bilateral frontal sinuses, ethmoidal sinuses, maxillary sinuses, and sphenoid sinuses (white arrows).

**FIGURE 2:**
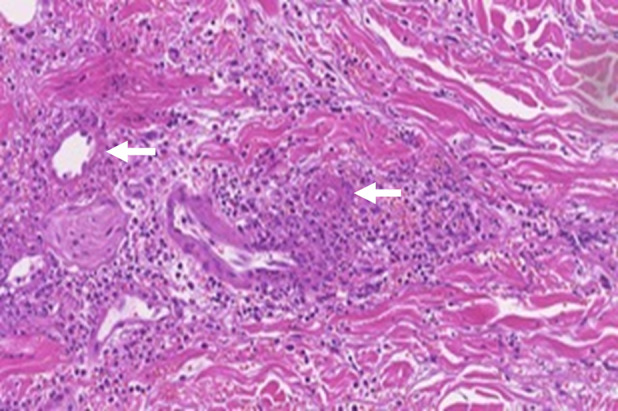
Histopathological findings of the skin biopsy (Haematoxylin and eosin staining, original magnification ×400). The skin biopsy revealed eosinophilic granulomatous inflammation and necrotizing vasculitis (white arrows).

A 79‐year‐old Japanese male with a history of refractory asthma presented with generalized purpura. He had started benralizumab 4 months earlier after 8 years of standard treatment, including oral corticosteroids. After starting benralizumab, his asthma was well‐controlled, and he reduced his prednisolone dose from 7.5 to 2 mg/day. On admission, he had a fever (38.6°C) and multiple purpura with erythema and hematoma on the extremities and trunk (Figure 1AB). Laboratory tests showed elevated levels of C‐reactive protein (11.02 mg/dL), D‐dimer (12.6 μg/mL), immunoglobulin E (4,841 U/mL) and rheumatoid factor (240 U/mL). Antinuclear antibody, PR3‐ANCA, and MPO‐ANCA assays were negative. Chest CT revealed bilateral pleural effusions (Figure 1C), and head CT showed findings consistent with sinusitis (Figure 1D). Prednisolone (1.0 mg/kg/day) was initiated after a skin biopsy. Eosinophils increased, reaching 6450/μL, and mononeuritis developed. The skin biopsy revealed vasculitis with extravascular eosinophilic infiltration and fibrinoid necrosis (Figure 2), suggesting EGPA. He met the 2022 American College of Rheumatology/European Alliance of Associations for Rheumatology Classification Criteria for EGPA^6^, with a score of 10 (obstructive airway disease +3; blood eosinophil ≥1×10^9^/L + 5; and extravascular eosinophilic‐predominant inflammation on biopsy +2), and was diagnosed with EGPA. Azathioprine and mepolizumab were added to corticosteroid therapy, leading to rapid improvement and sustained remission for over a year.

## DISCUSSION

Our patient is a very rare case of EGPA that developed associated with a reduction in oral steroids while receiving benralizumab treatment for severe asthma. It is also important to note that EGPA was completely controlled by the use of mepolizumab in addition to steroids and immunosuppressive agents.

In addition to our case, five other cases of EGPA have been reported while using benralizumab in the past (Table 1).^7^, ^8^, ^9^, ^10^ Our patient was 79 years old and the oldest of these patients (28–66 years old). In these patients, the time from benralizumab administration to the onset of EGPA ranged from 14 to 36 weeks, and the present case was within this range at 16 weeks. With the exception of the patient reported by Carrette et al., four of the patients had eosinophilia in the peripheral blood at the onset of EGPA, and this was also observed in our case. Two of the five previously reported cases of EGPA developed EGPA during or after oral corticosteroid drug reduction or discontinuation, and our patient also developed the condition during oral corticosteroid drug reduction.

**TABLE 1:** Case reports of EGPA during treatment with benralizumab.

Author, year	Age	Gender	Time from administration of benlalizumab to onset of EGPA (weeks)	Dosage of oral prednisolone: Before benralizumab administration (mg)	Dosage of oral prednisolone: After benralizumab administration (mg)	Maximum number of eosinophils in peripheral blood after benralizumab administration (/mm^3^)	MPO‐ANCA
Hocevar A, et al. (2020)^7^	28	Male	36	8	0	1310	Positive
Hocevar A, et al. (2020)^7^	58	Female	24	0	0	1410	Negative
Caminati M, et al. (2021)^8^	55	Female	14	N.A.	0	2800	Positive
Lim A, et al. (2021)^9^	31	Male	23	37.5	5	4600	Positive
Carrette A, et al. (2022)^10^	66	Female	N.A.	N.A.	N.A.	N.A.	N.A.
Our patient	79	Male	16	7.5	2	6450	Negative

*Note*: Summary of previously reported cases of EGPA during benralizumab use, including our case.

Abbreviation: N.A., not applicable.

The hypothesis that seems most plausible as a mechanism for the development of EGPA during benralizumab treatment is that a reduction in corticosteroid dose exposes latent EGPA. A further possibility is that pre‐existing EGPA has coincidentally evolved and entered a vasculitic phase, resulting in this presentation. That is, while benralizumab was effective in controlling the initial airways disease symptoms, there was breakthrough vasculitic phenomenon that occurred as the disease evolved. Indeed, the fact that EGPA developed during oral corticosteroid reduction in half of the six cases, the sum of the five previously reported cases and our patient, supports this hypothesis. It is also interesting to note that EGPA developed while using benralizumab, which may be effective in the treatment of EGPA, and was well controlled with mepolizumab, although only in the report by Caminati et al. and our case. The difference in the mechanism of action between benralizumab and mepolizumab, specifically whether binding to IL‐5 or the IL‐5 receptor and the presence or absence of antibody‐dependent cellular cytotoxicity (ADCC),^11^ may contribute to their different effects on EGPA. It has also been reported that IL‐5Rα expression is functionally reduced in lung tissue compared to circulating eosinophils,^12^ which may explain the mechanism by which EGPA develops during benralizumab administration.

In conclusion, this case emphasizes the importance of monitoring patients with severe asthma for EGPA development during benralizumab treatment, especially during oral corticosteroid reduction. Clinicians should be aware of this potential adverse event and promptly initiate appropriate management, which may include mepolizumab, corticosteroids, and immunosuppressive agents. Further studies are needed to elucidate the different mechanisms of action of mepolizumab and benralizumab in EGPA and to guide optimal management strategies.

## AUTHOR CONTRIBUTIONS

**Mitsukuni Sakabe**: Main author. **Kazunori Tobino**: Image selection; overview. **Yumi Obata**: Data collection. **Shota Sogabe**: Data collection and overview. **Kazuki Uchida**: Data collection and review. **Yosuke Murakami**: Review and overview. All authors discussed the results and contributed to the final manuscript.

## CONFLICT OF INTEREST STATEMENT

None declared.

## ETHICS STATEMENT

The authors declare that appropriate written informed consent was obtained for the publication of this manuscript and accompanying images.

## Data Availability

Data sharing is not applicable to this article as no new data were created or analyzed in this study.
